# Positional therapy in sleep apnoea - one fits all? What determines success in positional therapy in sleep apnoea syndrome

**DOI:** 10.1371/journal.pone.0174468

**Published:** 2017-04-13

**Authors:** Natascha Troester, Michael Palfner, Markus Dominco, Christoph Wohlkoenig, Erich Schmidberger, Martin Trinker, Alexander Avian

**Affiliations:** 1 Division of Pulmonology, Department of Internal Medicine, Medical University Graz, Graz, Austria; 2 Institute for Medical Informatics, Statistics and Documentation, Medical University Graz, Graz, Austria; 3 Ludwig Boltzmann Institute for Lung Vascular Research, Graz, Austria; University of Rome Tor Vergata, ITALY

## Abstract

**Introduction:**

Positional therapy is a simple means of therapy in sleep apnoea syndrome, but due to controversial or lacking evidence, it is not widely accepted as appropriate treatment. In this study, we analysed data to positional therapy with regard to successful reduction of AHI and predictors of success.

**Methods:**

All consecutive patients undergoing polysomnography between 2007 and 2011 were analysed. We used a strict definition of positional sleep apnoea syndrome (supine-exclusive sleep apnoea syndrome) and of therapy used. Patients underwent polysomnography initially and during follow-up.

**Results:**

1275 patients were evaluated, 112 of which had supine-exclusive sleep apnoea syndrome (AHI 5-66/h, median 13/h), 105 received positional therapy. With this treatment alone 75% (70/105) reached an AHI <5/h, in the follow-up 1 year later 37% (37/105) of these still had AHI<5/h, 46% (43/105) yielded an AHI between 5 and 10/h. Nine patient switched to APAP due to deterioration, 3 wanted to try APAP due to comfort reasons. At the last follow-up, 32% patients (34/105) were still on positional therapy with AHI <5/h. BMI was a predictor for successful reduction of AHI, but success was independent of sex, the presence of obstructive versus central sleep apnoea, severity of sleep apnoea syndrome or co-morbidities.

**Conclusion:**

Positional therapy may be a promising therapy option for patients with positional sleep apnoea. With appropriate adherence it yields a reasonable success rate in the clinical routine.

## Introduction

As frequently and recently stated [1, 2], positional therapy (PT) constitutes a simple means of therapy in a common variant of sleep apnoea syndrome (SAS) that nevertheless has failed to reach widespread use so far. On the one hand, this may be due to the fact that PT still does not seem to be accepted as efficient therapy, on the other hand compliance and comfort appear to be somewhat dubious. However, with supine position being predisposed for further breathing impairment, avoidance of such seems to be a straight-forward approach. As no standardized method exists, the possibilities are vast, ranging from tennis ball technique [3, 4] to backpacks [5] and sleep position trainer [6] with various outcomes, advantages and drawbacks. This and the also highly variable definition of what exactly comprises positional SAS [7, 8, 9, 10], add to the confusion in this field and hence to a lack of acceptance in the treating professionals. We therefore analysed patients using a strict definition of positional SAS, but nevertheless making allowance to the fact that an appropriate PT must be an individual device. The aim of our study was to determine efficacy and efficiency of PT as well as to identify predictors of success.

## Methods

Patients presenting to the sleep laboratory at the Medical University Graz, Department of Pulmonology, from 2007 to 2011 were analysed with regard to positional dependency of sleep apnoea syndrome. Data derived from clinical routine had been prospectively entered in a database with patients´ written consent. Decision to positional treatment rather than PAP-therapy was made according to results of the PSG regarding positional predominance of respiratory events. The study was approved by the local ethics committee at the Medical University of Graz.

The first PSG was performed to establish the diagnosis. If respiratory events (apnoeas and hypopnoeas) occurred exclusively in supine position (AHI <5/h in all other positions) as proven by position sensor corrected with video surveillance (supine-exclusive SAS), mechanical PT was initiated. No minimal time lateral left and right, prone or supine was required, but the staff tried to obtain data from sleep in all positions. This guaranteed supine position in every patient, but sufficient time in all positions was not obtained in all patients. Patients were instructed to use tightly fitted backpacks filled with big items such as footballs, swimming aids or long plastic pipes or commercially available models such as Somnocushion^®^. PT hence consisted of variations of these recommendations. Most patients used backpacks stuffed with balls or other equipment and adapted them according to their own body structure and comfort. No support by any of the companies providing positional therapy or training devices was obtained.

Shirts with tennis balls or other small items were not used. PT was initiated during an overnight stay with PSG one to six months after diagnosis depending on logistics in the sleep laboratory. First follow-up was within the first year of treatment. The last follow-up or therapy change took place in the course of the second year of treatment. Only the results of patients who had overnight PSG at all times are presented in this analysis.

PSG examination was conducted using a standard montage according to AASM [11]. PSG examination was conducted using a standard montage for electrical activity of the brain (C3–A2, C4–A1, O1-A2, O2-A1, F3-A2, F4-A1), eye movements, submental EMG, combined anterior tibialis EMG for leg movements, ECG, and respiration (using an oronasal thermal airflow sensor and a nasal pressure transducer as well as abdominal and thoracic RIP belts), pulse oxymetry, blood pressure and video. Polysomnograms were initially scored by a polysomnographic technologist and examined by a sleep specialist. Polysomnography was performed using a digital device (Somnomedics^®^, Germany, 2.4.0 Domino^®^ from September 2010 to 2011, Harmonie-S, Version 6.1c DE-0, Schwarzer Comlab44^®^ was used from September 2007 to September 2010). Polysomnograms were scored by a polysomnographic technologist and examined by two different sleep specialists. Respiratory events and sleep staging were scored according to the rules of the AASM [11], OSAS was diagnosed with AHI >5/h. For hypopnoeas, a drop of amplitude of at least 30% accompanied by at least 3% desaturation or arousal was used as definition. RERAs were scored via nasal pressure transducer as elevated breathing effort or flattening accompanied by an arousal. Sleep apnoea syndrome was defined as obstructive sleep apnoea syndrome (OSAS) if >50% of the respiratory events were obstructive, as central sleep apnoea syndrome (CSAS) in case of >50% central respiratory events and as combined if both types of apnoeas were present, but hypopnoeas were predominantly obstructive.

To characterize patients with successful and non-successful PT, patients were grouped according to their post treatment full-night AHI (successful PT: post treatment AHI <5, non-successful PT: post treatment AHI ≥ 5). A model relating the patient’s post treatment full-night AHI (successful / non-successful) to baseline characteristics was built using logistic regression. In the first step univariate logistic regression analyses were performed. Significant variables or variables which showed a specific tendency were selected for multivariate logistic regression. Variables in the final model were selected with a forward stepwise procedure. The decision to remove variables was based on a likelihood-ratio test. These analyses were also done for both sexes separately. Furthermore baseline characteristics of two entities of sleep apnoea syndrome (OSAS, CSAS) were compared using t-Test or Mann Whitney U-Test, as appropriate. For categorical variables Chi square Test of Fisher’s exact Test was used. Statistical analyses were performed using SPSS 22.0 (SPSS, Chicago, IL, USA). Continuous data are presented as median and interquartile range or mean and standard deviation. Categorical data are presented as absolute and relative frequencies.

## Results

### Overall results

**Fig 1:**
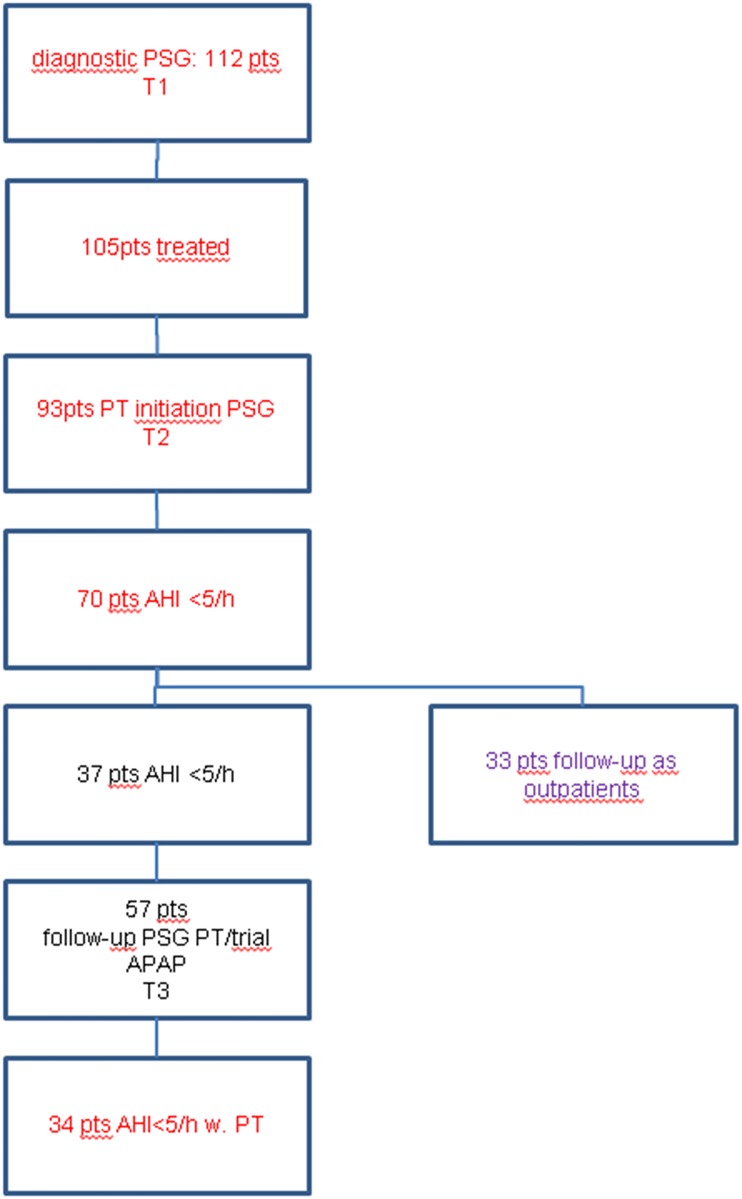
Procedure and therapy.

**Fig 2:**
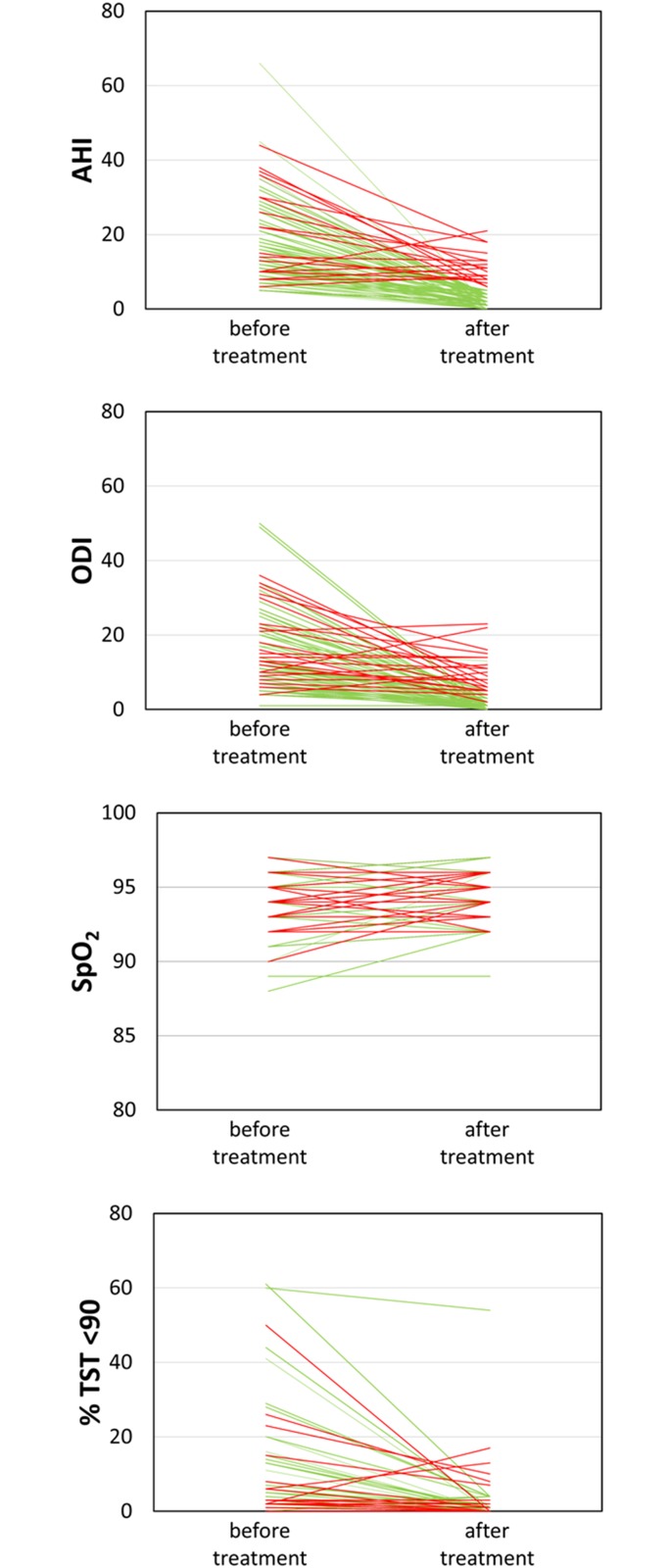
Overall results, green line: Treatment AHI<5/h, red line: Treatment AHI≥5/h.

Of a total of 1275 patients referred to the sleep lab, 112 patients were diagnosed with positional SAS (T1, see Fig 1) according to our definition (supine-exclusive SAS), which comprises 8.8% of all examined patients. Seven patients had a normal AHI (<5/h) and were not treated at this time. The remaining 105 patients showed an AHI between 5/h and 66/h (median 13/h). Baseline characteristics of these patients are shown in Table 1. In 93 of these patients, PT was initiated during an overnight stay with PSG. Twelve patients were instructed how to use PT and did not undergo another PSG. Of the 93 patients who underwent treatment initiation with an overnight stay with PSG (T2), 70 patients (75%) achieved an AHI <5/h via PT, 5 had an acceptable reduction (AHI 5-10/h) with PT and did not want APAP. AHI ≥ 5/h with PT was either due to an increase of lateral AHI which was not seen initially as patients had slept in the supine position only (13 patients) or due to more time in REM with emerging respiratory events (10 patients). Weight change did not play a role (p = 0.426). Regarding all 93 patients, AHI with PT lay between 0 and 21/h (median 2/h). Overall, with PT all important sleep related breathing parameters could be improved (Fig 2). Long term (within one year of treatment initiation), 34 patients (37%) still had an AHI <5/h and felt comfortable with PT. Twelve patients had to swap treatment to APAP, 9 of which due to an increase of AHI (in lateral position), 3 wanted to try APAP because of lack of comfort with PT, only one of these stuck to APAP, one finally continued with PT, one declined all treatment. One patient improved due to weight loss, one patient switched from APAP to PT for comfort reasons. Nine patients had an AHI of 5-10/h, but did not want to use APAP. Detailed information is provided in S1 Fig.

**Table 1:** Characteristics of patients receiving treatment.

n = 105	Mean	SD	Min	Max
Age (years)	57.9	13	24	83
Height (cm)	170.8	8.9	149	189
Weight (kg)	85	12.3	59	117
BMI	29	3.3	22	39
AHI (/h)	16.3	10.8	5	66
RDI (/h)	20.4	9.1	8	44
ODI (/h)	14.2	10	1	50
SpO2 min %	83.4	4.8	66	92
TST<90%SpO2 (%)	6.9	12.5	0	61

### Change of treatment

Change to APAP in the long run was either due to lack of comfort or increase of AHI despite PT. This was due to an increase of lateral AHI, partially because of REM, but REM did not change significantly (overall p = 0.678, success group p = 0.088, non-success group p = 0.136).

Of all patients who were seen in the last follow-up (T3), 43 of 105 initial PT patients (46%) still had at least acceptable results (AHI ≤10/h), 34 of 105 initial PT patients (37%) still showed a normalization of AHI (AHI ≤5/h).

### Success vs. non-success

In univariate analysis the only predictor for successful PT was BMI (p = 0.029; OR 1.18 95%CI 1.02–1.36). Furthermore diastolic blood pressure (p = 0.080, OR 1.04 95% CI 1.00–1.08) showed a tendency. In multivariate analysis only BMI remained significant. Therefore, severity of SAS does not exclude PT as possible treatment, nor does weight provided that the device matches the patients`height. Co-morbidities do not necessitate positive airway pressure therapy a priori either, as none of the analysed parameters yielded any statistical significance (Table 2).

**Table 2:** Univariate and multivariate predictors for success with PT.

Parameter	success with PT	non-success with PT	univariate	multivariate	OR (95%CI)
	(AHI <5/h)	(AHI ≥5/h)			
Age	59 ± 13	59 ± 11	0.914		
**BMI**	**28 (26–30)**	**31 (29–33)**	**0.029**	**0.029**	**1.18 (1.02–1.36)**
Height	171 ± 9	167 ± 5	0.104		
Blood pressure syst	136 (126–151)	139 (132–157)	0.647		
Blood pressure diast	87 ± 12	92 ± 13	0.080		
Heartrate	65 (59–74)	71 (63–77)	0.203		
FEV1	3.0 (2.5–4.0)	3.0 (3.0–3.0)	0.228		
VCmax	4.0 (3.0–5.0)	4.0 (4.0–5.0)	0.864		
DLCO/VA	97 (87–111)	98 (89–116)	0.298		
Haemoglobin	15 (14–15)	14 (13–16)	1.000		
Cholesterin	202 ± 42	192 ± 43	0.322		
Triglycerides	122 (90–179)	125 (95–183)	0.843		
HbA1c	37 (34–39)	38 (34–42)	0.936		
NTproBNP	58 (27–153)	59 (28–194)	0.903		
Glomerular filtration rate	76 ± 18	76 ± 19	0.967		
Arousalindex	14 (9–19)	15 (11–20)	0.451		
Oxygen desaturation index	11 (7–20)	15 (10–23)	0.185		
TST<90%Sat	397 (346–426)	384 (327–422)	0.972		
Respiratory disturbance index	21 ± 9	30 ± 13	0.195		
Periodic limb movement index	5 (1–29)	12 (3–22)	0.660		
Diabetes mellitus	11.6%	8.7%	0.700		
COPD	1.7%	10%	0.156		
Atrial fibrillation	8.7%	13.0%	0.546		
Hypertension	56.5%	69.6%	0.272		
Chronic renal dysfunction	5.7%	8.7%	0.616		
Coronary artery disease	8.6%	13.0%	0.532		
Apoplexy	4.3%	0.0%	0.999		
Co-morbidities overall	58.6%	69.6%	0.350		

For each continuous variable mean and SD or median and IQR is given and for each categorical variable the relative number is given.

### Results according to sex

**Fig 3:**
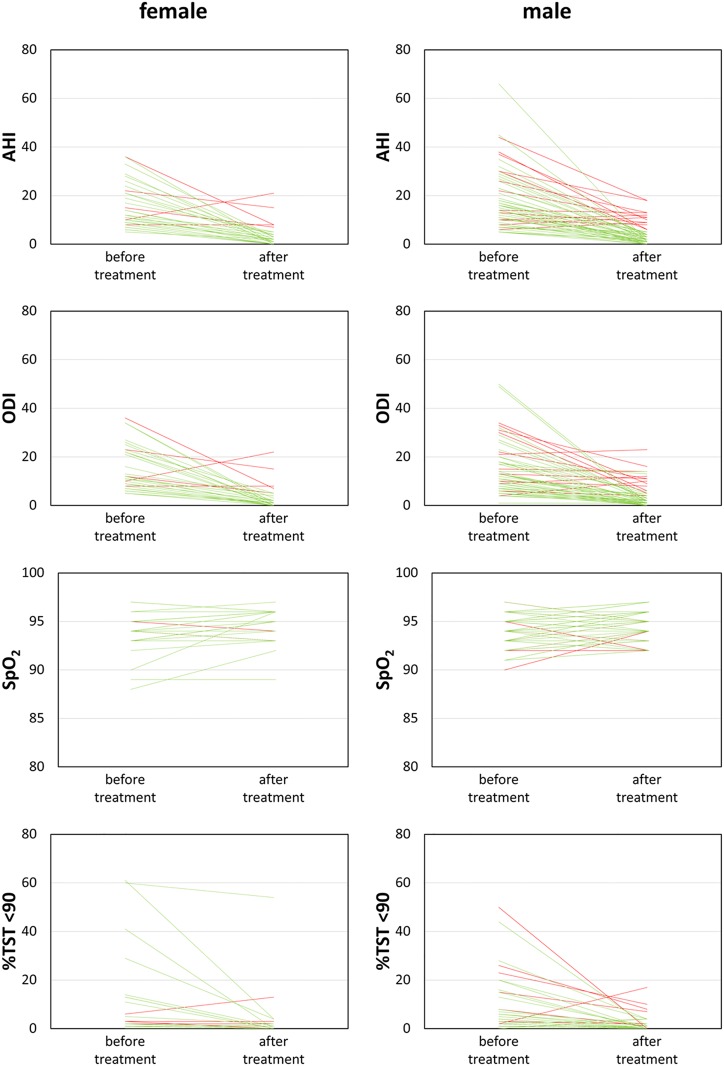
Results male/female patients, green line: Treatment AHI<5/h, red line: Treatment AHI≥5/h.

Of the 105 patients receiving treatment, 35 (33.3%) were female. They were older than the male patients (66, 55–73; vs 55, 46–63 years, p<0.001). They were also smaller in height (163, 158–169; vs 173.8, 170–179 cm, p<0.001) and weight (74, 67–86; vs 87, 80—95kg, p<0.001) without difference in BMI (29, 27–32; vs 29, 26–31, p = 0.569). Details on differences are outlined in Table 3. AHI at diagnosis (p = 0.734) and follow-up (p = 0.251) and its change (p = 0.656) was similar to that of male patients (See Fig 3). Predictors for success were comparable to the overall results with a significant influence of height in males (p = 0.001, OR 0.72 95% CI 0.60–0.87) and weight in females (p = 0.037, OR 1.12 95% CI 1.01–1.25).

**Table 3:** Differences between female and male patients.

	sex	
	female	male
Age	66 (55–73)	55 (46–63)
BMI	29.4 ± 3.9	28.8 ± 3.0
Height	163.2 ± 6.8	174.6 ± 7.3
BPsyst	136 ± 21	142 ± 18
BPdiast	85 ± 13	90 ± 12
Heartrate	68 (61–71)	67 (61–75)
FEV1	2 (2–3)	3 (3–4)
VCmax	3 (3–4)	5 (4–5)
DLCO/VA	88 (80–92)	106 (91–115)
Hb	13 (12–14)	15 (14–16)
Cholesterol	204 (171–236)	198 (162–231)
Triglycerides	119 (98–143)	127 (92–183)
HbA1c	38 (35–41)	37 (34–38)
NT-BNP	125 (84–459)	36 (19–96)
GFR	67 ± 21	81 ± 14
ArII	12 (8–19)	16 (11–20)
ODI	11 (7–22)	11 (7–18)
TST	384 (327–428)	388 (344–420)
RDI	21 ± 8	20 ± 10
PLMS/h	16 (2–39)	4 (2–20)
Co-morbidities	77.1%	51.4%

### Results according to various entities of sleep apnoea syndrome

**Fig 4:**
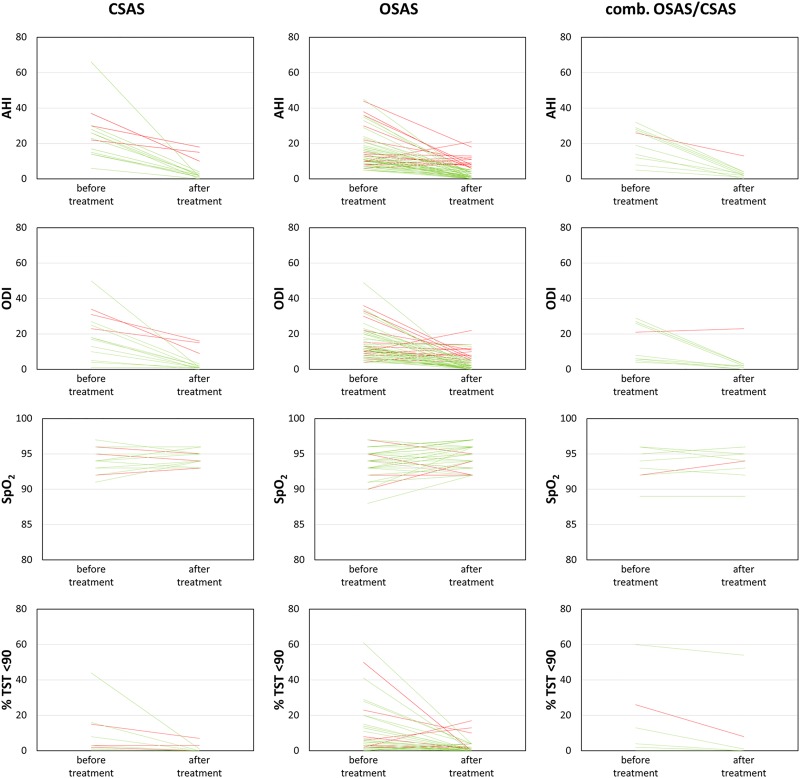
Results OSAS/CSAS, green line: Treatment AHI<5/h, red line: Treatment AHI≥5/h.

Of the 105 patients receiving treatment, 76 patients (72.3%) were identified as OSAS, 16 (15.2%) as CSAS, 13 (12.3%) as combined SAS. As OSAS and CSAS represent the more distinct entities, only results of these two are presented here: Patients with CSAS were taller than OSAS patients (175.5, 172.0–179.5cm; vs. 169, 162.7–174.5cm, p = 0.015), had initially a higher median AHI (22.5, 14.0–29.0/h; vs. 11.0, 8.0–17.5/h, p = 0.009) and a higher median SpO_2_ (86, 84.5–89.5%; vs. 83.0, 81.0–86.0%, p = 0.002). With therapy, both groups ended with similar results (median AHI 2, 1–4; vs. 2, 0–5/h both, p = 0.676; median SpO_2_ 90, 87–91% in CSAS; 89, 86–91% in OSAS, p = 0.39, see Fig 4).

## Discussion

Previous trials concerning positional SAS and treatment yielded heterogeneous results due to different definitions of positional SAS itself and due to variable treatment approaches. Our study showed initial success of PT in 75% of patients with an appropriate device with a long-term change to PAP therapy of only 2%. We defined positional SAS only in cases with events appearing exclusively in supine position as detected by positional sensor and manual correction using video surveillance. We therefore follow the definition of supine-isolated SAS as introduced by Joosten *et al*. [10]. We consider this the only appropriate definition of positional SAS. Including supine-predominant SAS with AHI non-supine up to 50% of supine AHI skews the results of the efficacy of PT as only a part of the events is addressed with this method. Thus, if positional SAS is defined strictly, the prevalence is lower than found in previous studies [10, 12].

Severity of SAS was not a predictor for failure in our study. Although BMI was a predictor for success, an improvement was shown in most patients. In male patients, height played a greater role, in female ones it was weight. Therefore, severe SAS as well as obese patients can be treated successfully with PT which is in contrast to previous findings [13]. However, as BMI was a predictor of success, obese patients might need a tighter follow-up and the use of an appropriately large device must be emphasised. The difference to the approach in previous studies is that we tried to use an appropriate tool, i.e. large and big enough for their body structure. In the follow-up, this therapy remained successful in the majority of the patients. Thus, an appropriate device that is large enough to sufficiently prevent supine position and to stabilize head position (which was not analysed separately in this study) allows a high success rate of PT. These results were true also in gender related analysis, although the influence of weight and BMI were different between male and female patients. However, the female patients group was considerably smaller than the male patients group which might have led to skewed results. The type of sleep apnoea syndrome did not influence success or non-success, either. PT was successful in OSAS and CSAS alike. This confirms previous hypotheses and results according to which CSAS also shows position dependency and response to PT. Upper airway instability seems to play a role in CSAS also, thereby evoking apnoeas, hypopnoeas, and pronounced desaturation in supine position. Thus, an increase in hypoxic drive, together with an increased sensitivity of chemoreceptors may promote further respiratory instability. In addition, reduction of lung volumes and oxygen stores in supine position seem to play a role [14, 15, 16, 17].

As for change of treatment, deterioration was not the only reason, but lack of comfort with PT was another factor, but change from APAP to PT due to comfort reasons occurred as well.

### Limitations

This study was conducted as a retrospective analysis of data and is therefore prone to confounding. However, the data had been prospectively collected in a comprehensive database and were based on polysomnography rather than polygraphy. Due to organisational changes, not all patients had a follow-up with an overnight stay. Therefore, fewer PSG data exist for the final follow-up than initially intended, so a higher success rate long term is possible. We did not compare positional SAS patients with non-positional ones or with results from APAP treatment as the study was designed only to analyse success rates of PT per se and possible predictors, so no data are presented in comparison. However, our data still show a wide range of weight and SAS severity without predominantly lean patients or lower severity underlining our hypothesis of efficacy of PT in all sorts of patients. However, we only analysed data derived from overnight stays with PSG in hospital, so we have no information regarding success at home or adherence. Although the staff tried to have patients sleep in all positions, the main focus was to obtain supine position, so the percentage of other positions varied or occurrence of all positions apart from supine could not be guaranteed. Individual variability of AHI, as repeatedly discussed and assumed to be caused by body fluid shift, sleep consolidation, and first night-effect [18,19], also could have played a role in the “progress” and change of results in the follow-up. As for comfort with PT, only one patient changed his treatment to APAP due to comfort reasons (see flowchart in supplement).

## Conclusion

Positional therapy could comprise an effective means of treatment independent of severity of sleep apnoea syndrome, obesity, and co-morbidities. However, an adequate form of positional therapy related to patients´ body structure must be used. This does not necessarily have to be a commercially available device, but in most cases a handmade version by the patients themselves proves to be effective due to personalized size and design. Supine-exclusive positional sleep apnoea enhances the probability of success in contrast to more liberal definition of positional sleep apnoea. However, day-to-day variability and change or progress of the initial results must be taken into account, and follow-up is needed.

## Supporting information

S1 Fig Study procedure flowchart. (TIF)

## Abbreviations

AHI: apnoea-hypopnoea-index
APAP: automatically titrated positive airway pressure
ARlI: Arousal index
BMI: body-mass-index
BP: systemic blood pressure
CSAS: central sleep apnoea syndrome
ODI: oxygen desaturation index
OSAS: obstructive sleep apnoea syndrome
PAP: positive airway pressure
PT: positional therapy
RDI: respiratory disturbance index
REM: rapid eye movement (sleep)
SAS: sleep apnoea syndrome
TST<90%: total sleep time with oxygen saturation <90%
