# Supplementary figures and images for: Positional therapy in sleep apnoea - one fits all? What determines success in positional therapy in sleep apnoea syndrome
Source: PLoS One. 2017 Apr 13;12(4):e0174468. doi: 10.1371/journal.pone.0174468 (PMC5390972; doi:10.1371/journal.pone.0174468)

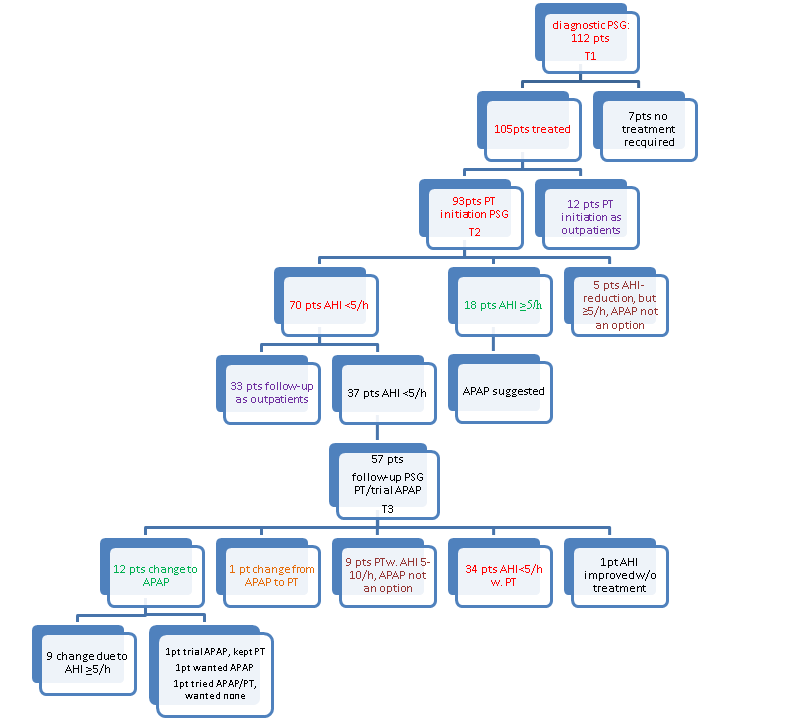

Supplement: S1 Fig — (TIF) [file pone.0174468.s001.tif]
